# *Strongylocentrotus nudus* Eggs Polysaccharide Enhances Macrophage Phagocytosis Against *E.coli* Infection by TLR4/STAT3 Axis

**DOI:** 10.3389/fphar.2022.807440

**Published:** 2022-03-17

**Authors:** Xinlei Tian, Min Guo, Xiaoya Zhang, Lingfeng Guo, Nan Lan, Yaojun Cheng, Yannan Han, Mingxin Wang, Zhonglu Peng, Changlin Zhou, Hongye Fan

**Affiliations:** ^1^ School of Life Science and Technology, China Pharmaceutical University, Nanjing, China; ^2^ School of Pharmacy, Xiangnan University, Chenzhou, China

**Keywords:** SEP, macrophage, TLR4, phagocytosis, infection

## Abstract

Antibiotics resistance is one of the most significant public health threats globally. Strategies that strengthen host defenses to control pathogen infection has become a hot research field. Macrophages are part of early host defense mechanisms, and are activated *via* host pattern recognition receptors (PRRs), such as Toll-like receptor 4 (TLR4), which then facilitates phagocytosis and elimination of invading pathogens. However, few activators of PRRs have been approved for clinical use because of their toxic effects. This study aimed to investigate whether *Strongylocentrotus nudus* eggs polysaccharide (SEP), a non-toxic extract from seafood, contributes to host defense against bacterial infection. Results showed that SEP promoted bacterial clearance by enhancing phagocytosis by macrophages during *E. coli* infection *in vitro*, but was inhibited by TLR4 specific inhibitor TAK-242, STAT3 inhibitor Stattic or blockade of CD64. In addition, SEP protected mice from *E. coli* induced mortality, reduced pulmonary inflammation and inhibited dissemination of bacteria to organs, while TAK-242 retarded the protection of SEP. Overall, SEP strengthened innate host defense and improved the outcome in bacterial infection, suggesting that SEP could be used as a potential immunomodulator in host-directed therapies.

## Introduction

Bacterial infectious diseases remain a serious global health threat, especially for patients in immunocompromised status (Rudd et al., 2020). Sepsis is a life-threatening organ dysfunction caused by a dysregulated host response to infection, and is one of the major causes of mortality in ICUs (Hatfield et al., 2018). Although antibiotics are the primary treatment for bacterial infection, excessive clinical use of antibiotics is the main cause of the rapid increase in the prevalence of antibiotic-resistant bacteria and hospital-acquired infections (Laxminarayan et al., 2016; Rudd et al., 2020). However, the host’s innate immune surveillance is the first line of defense against bacteria, playing a crucial role in governing the bacterial infection and stopping the transmission of resistant strains between patients (Zumla et al., 2016; Kaufmann et al., 2017). Therefore, there is a growing need to develop novel pharmaceutical interventions, and strategies that strengthen innate host defenses against bacterial infection have recently gained more attention.

Macrophages are professional phagocytes of innate immunity, provide protection against infections by effective phagocytosis of bacteria and maintain tissue homeostasis (Bah and Vergne, 2017); macrophages depend on a series of pattern recognition receptors (PRRs) to phagocytose bacteria (Brubaker et al., 2015). Toll-Like Receptor 4 (TLR4), one of the highly conserved PRRs, plays a crucial role in host defense by enhancing macrophage phagocytosis and bacterial clearance (Hernandez et al., 2019). Without functional TLR4, both human or mouse are more susceptible to pathogenic bacterial infection (Bihl et al., 2003; Branger et al., 2004; O'Brien et al., 2005). Excessive stimulation of TLR4 by lipopolysaccharide (LPS) not only enhances macrophage phagocytosis of bacteria, but also induces overwhelming proinflammatory cytokines production which can lead to multiple organ failure and death of sepsis (Rice et al., 2010; Hernandez et al., 2019). Thus, two TLR4 antagonists TAK-242 and Eritoran reached clinical trials as anti-sepsis agents, unfortunately both failed to suppress cytokine levels or improve the survival of septic patients (Rice et al., 2010; Anwar et al., 2019). Interestingly, a series of mild non-toxic agonists of TLR4 such as MPLA, have been proved to enhance host defense against infection *via* macrophage phagocytosis, and reduce systemic inflammation (Fensterheim et al., 2018; Hernandez et al., 2019). These findings suggested that innate host defense against infection can be enhanced by mild activation of TLR4, and the mild agonists of TLR4 are potential candidates in host-directed therapies.

Fcγ receptors (FcγRs) are a kind of PRRs necessary for macrophages to recognize and phagocytose bacteria (Aderem and Underhill, 1999). In mouse macrophages, FcγRI (CD64), FcγRIIB (CD32), and FcγRIIIA (CD16) are constitutively expressed (Aderem and Underhill, 1999; Weighardt et al., 2000; Nimmerjahn and Ravetch, 2008). Of note, CD64 had been shown to mediate the internalization of enteropathogenic *E. coli* in the absence of opsonins (Vallieres and Girard, 2017). And macrophages with high expression of CD64 are responsible for phagocytosis and killing of pathogen (Danikas et al., 2008). Furthermore, the expression level of CD64 in macrophages of sepsis survivors was higher than non-survivors (Liu et al., 2007). Nevertheless, few agents targeting FcγRs have been reported to regulate macrophage phagocytosis.

*Strongylocentrotus nudus* eggs polysaccharide (SEP) is homogeneous polysaccharide components isolated and purified from *Strongylocentrotus nudus*, containing an α-1,4-linked backbone and α-1,6-linked branches (Ke et al., 2014). *Strongylocentrotus nudus*, also called the Dalian purple sea urchin, is a major economic species and widely distributed in China. And their eggs are favorite seafood for pleasurable taste and high nutrition. According to traditional Chinese medicine, sea urchins can alleviate precordial pain and enhance immunity (Shang et al., 2014). Previous studies have shown SEP exerts anti-cancer effects *via* augmenting T cell and NK cell proliferation and cytotoxicity (Xie et al., 2017; Hu et al., 2018; Xie et al., 2019). Moreover, SEP attenuated cyclophosphamide-induced myelosuppression and activated macrophages by increasing the production of nitric oxide (NO) (Liu et al., 2008). However, there is no report on the pharmacological role of SEP in innate immunity against bacteria.

To elucidate the biological and pharmacological roles of SEP on the phagocytic activity of macrophages in host defense against bacteria, the models of *E. coli* infected mice were introduced. The results revealed that SEP protected against *E. coli-*induced sepsis and augmented macrophages phagocytosis *via* TLR4 signaling. Moreover, SEP promoted macrophages filopodia formation and phagocytosis by activating TLR4/STAT3/CD64 signals. Taken together, this study indicated SEP could be a potential immunopotentiator for innate host defense against bacterial infections.

## Materials and Methods

### Chemicals and Reagents

PHrodo™ Green *E. coli* BioParticles™ was purchased from Thermo Fisher Scientific (Waltham, MA, United States). Stattic, Fludarabinum, C29, TAK-242 were bought from MCE (Monmouth Junction, NJ, United States). BAY 11-7085, SP610025, PD184352, LY294002, AZD0530 and Y-27632 were purchased from Selleckchem (Houston, TX, United States). Mouse IL-1β, IL-6, TNF-α Quantikine ELISA Kit, Cytochalasin D were bought from R&D Systems (Minneapolis, MN, United States). Mouse myeloperoxidase/MPO ELISA Kit was purchased from MultiScience (Lianke) Biotech Co., Ltd. (Hangzhou, China). p-STAT3 (Tyr705), p-STAT1 (Tyr701), p-p65(Ser536), p-Src (Tyr416), p-Lyn (Tyr507), p-SAPK/JNK (Thr183/Tyr185), p-p38 (Thr180/Tyr182), p-ERK1/2 (Thr202/Tyr204) were bought from Cell Signaling Technology (Beverly, MA, United States). STAT3, STAT1, p65, Src, Lyn, JNK, p38, ERK1/2 were purchased from wanleibio (Shenyang, China). β-actin was purchased from Bioworld Technology (Bloomington, MN, United States). FITC anti-mouse F4/80 antibody (clone BM8), APC anti-mouse CD64 (FcγRI) antibody (clone X54-5/7.1), PE anti-mouse CD16/32 antibody (clone 93), purified anti-mouse CD16/32 antibody (clone 93) and Ultra-LEAF™ Purified anti-mouse CD64 (FcγRI) antibody (Clone W18349F) were purchased from Biolegend (San Diego, CA, United States). Rhodamine phalloidin and DAPI were purchased from Beyotime (Nanjing, China).

### Preparation of *Strongylocentrotus nudus* Eggs Polysaccharide

*Strongylocentrotus nudus* were purchased from Dalian Haibao Fishery Co., Ltd., a commercial breeding base in Dalian, China. SEP was extracted from *Strongylocentrotus nudus* eggs according to our previously reported method (Liu et al., 2007) and the lyophilized SEP powder was stored at −20°C in our laboratory (School of Life Science and Technology, China Pharmaceutical University, Nanjing, China). Briefly, *Strongylocentrotus nudus* eggs were kept frozen at −20°C. The crude, water-soluble polysaccharides isolated from these eggs were sequentially purified *via* Cellulose DEAE-52 and Sephacryl S-400 to yield the main fraction of SEP for subsequent analysis. High performance liquid chromatography (HPLC) system equipped with a TSKgel 4000 PWXL column and a Waters 2414 refractive index detector (Sigma–Aldrich, United States) was used to assess the purity of SEP. The HPLC profile showed a single symmetrical, sharp peak, and the content of SEP was 98.8% (Xie et al., 2019). SEP consisted of only glucose monomers with a mean molecular weight of 1.95 × 10^6^ Da. The structure of SEP was elucidated in our previous study (Liu et al., 2007). SEP has a main chain of α-(1→4)-D-glucan and a single α-D-glucose at the C-6 position every nine residue, on average, along the main chain (Liu et al., 2007; Liu et al., 2008; Wang et al., 2011; Ke et al., 2014).

### Mice

Male ICR mice (20-22 g, 5-6 weeks) were purchased from Yangzhou University (Yangzhou, China) and acclimatized for 1 week before molding. All animal experiments were in strict accordance with the protocols of the Ethics Committee of China Pharmaceutical University (Nanjing, China). The use and experiment of mice were performed according to the Laboratory Animal Care & Use Committee at China Pharmaceutical University.

### Bacteria and Cell Culture

*Escherichia coli* (*E.coli*) ATCC25922 was purchased from the American Type Culture Collection (ATCC). *E. coli* was grown to mid-exponential phase, then centrifuged, washed and diluted in PBS. RAW264.7 cells were purchased from China Center for Type Culture Collection (CCTCC, Shanghai, China) and cultured in DMEM medium (GIBCO, Beijing, China) with 10% fetal bovine serum (GIBCO, NY, United States) at 37°C in a humidified environment with 5% CO_2_. RAW264.7 cells were pre-treated with SEP (100 μg/ml) for 24 h. For inhibitor experiments, cells were pre-treated with chemical inhibitors for 2 h prior to SEP treatment. For the FcγRs blocking experiment, RAW264.7 cells were first stimulated with SEP for 24 h and then administrated with anti-mouse CD16/32 (2 μg/ml) or anti-mouse CD64 antibodies (2 μg/ml) for 1 h prior to *E. coli* infection.

### Mouse Model of *E.coli* Infection and Drug Administration

ICR mice were first pre-treated with SEP or vehicle (saline) by injection (25 mg/kg, i. v.) every 3 days for three times before sacrifice or *E. coli* infection. To determine the effect of SEP on mortality, mice from each group were administered a lethal dose of *E. coli* (9 × 10^8^ CFU, i. p.). To determine the therapeutic effect of SEP, mice from each group were injected with a lower dose of *E. coli* (3 × 10^8^ CFU, i. p.) and sacrificed at 18 h post-infection. Moreover, the TAK-242 group mice were pre-treated with TAK-242 (10 mg/kg, i. p.) 2 h before administration of SEP. Colony-forming units (CFU) of *E. coli* in tissue homogenates of lung, spleen, liver, and peritoneal lavage fluid were detected. For histopathological analysis, the right lung tissues were fixed in 4% paraformaldehyde fix solution (Beyotime, Nanjing, China), and hematoxylin and eosin (H&E) staining were conducted.

### Gentamicin Protection Assay

Gentamicin protection assay was used to determine the number of live intracellular bacteria during infection (Sharma and Puhar, 2019). Briefly, RAW264.7 cells were infected with *E. coli* with an MOI of 10 for 1 h at 37°C in antibiotic-free DMEM. After infection, cells were gently washed with cold PBS to stop bacteria internalization. To eliminate extracellular or membrane-bound bacteria, cells were treated with gentamicin (100 μg/ml) for 30 min. Then cells were lysed in Triton X-100 (0.04% vol per vol) and plated with serial dilution on LB plates for bacterial enumeration.

### Phagocytosis of pHrodo-Conjugated *E. coli* Bioparticles

For pHrodo-conjugated *E. coli* bioparticles phagocytosis, two detection methods were used. RAW264.7 cells were incubated with pHrodo-conjugated *E. coli* bioparticles with an MOI of 50 for 1 h at 37°C in a humidified chamber. Then cells were washed and fluorescence was read on a SpectraMax i3 spectrophotometer plate reader (Molecular Devices, CA, United States). Phagocytosis was calculated by subtracting the baseline fluorescence from wells containing pHrodo-conjugated *E. coli* bioparticles without RAW264.7 cells. Or cells were collected and analyzed using FACS Calibur flow cytometer (BD Biosciences, CA, United States). Phagocytosis was expressed as a percentage of positive macrophages over total cells counted, and histograms were generated using FlowJo software (Treestar, Inc., San Carlos, CA, United States).

### Flow Cytometry

RAW264.7 cells or peritoneal macrophages were labeled with FITC anti-mouse F4/80 antibody, APC anti-mouse CD64 antibody and PE anti-mouse CD16/32 antibody. Labeled cells were analyzed on FACS Calibur flow cytometer and data were analyzed using FlowJo software.

### Quantitative Real-Time PCR

Total RNA was extracted by Trizol reagent (Vazyme Biotech, Nanjing, China) and used HiScript® II QRT SuperMix (Vazyme, Nanjing, China) to generate cDNA. qRT-PCR was performed on the QuantStudio™ 3 Real-Time PCR System (Applied Biosystems, CA, United States) using SYBR green dye (Vazyme Biotech, Nanjing, China). The mRNA levels of all samples were normalized to GAPDH. The relative mRNA level of specific gene expression was analyzed by the method of 2^−ΔΔ^Ct.

### Western Blot

The method of western blot was performed as previously described (Pan et al., 2018). Briefly, lung tissues or RAW264.7 cells were lysed with RIPA lysis buffer with the protease inhibitor cocktail (Beyotime, Shanghai, China). Protein concentrations were determined by the BCA assay kit (Beyotime, Shanghai, China). Proteins were separated by SDS-PAGE and electrotransferred onto PVDF membranes. Protein bands were detected by Enhanced Chemiluminescent Substrate Kit (YEASEN, Shanghai, China) and imaged with Tanon 1600 (Tanon, Shanghai, China).

### Measurement of Cytokines

The lung tissues were weighed and homogenized with cytokine lysis buffer on ice. After vortex mixing, the mixtures were incubated at 4°C for 30°min, then centrifuged at 12000 rpm for 15 min. The supernatant was collected and frozen at −80°C. The cytokines levels were detected by ELISA kits according to the manufacturer’s protocol.

### Statistical Analysis

All data were presented as the mean ± SEM. Statistical significance was determined by unpaired Two-tailed Student’s *t*-test or by One-way ANOVA (GraphPad Prism 8). *P* values were used to determine statistical significance. *p < 0.05, **p < 0.01, ***p < 0.001, ****p < 0.0001.

## Results

### SEP Protects Against *E. coli* Infection by Enhancing Host Defense

**FIGURE 1:**
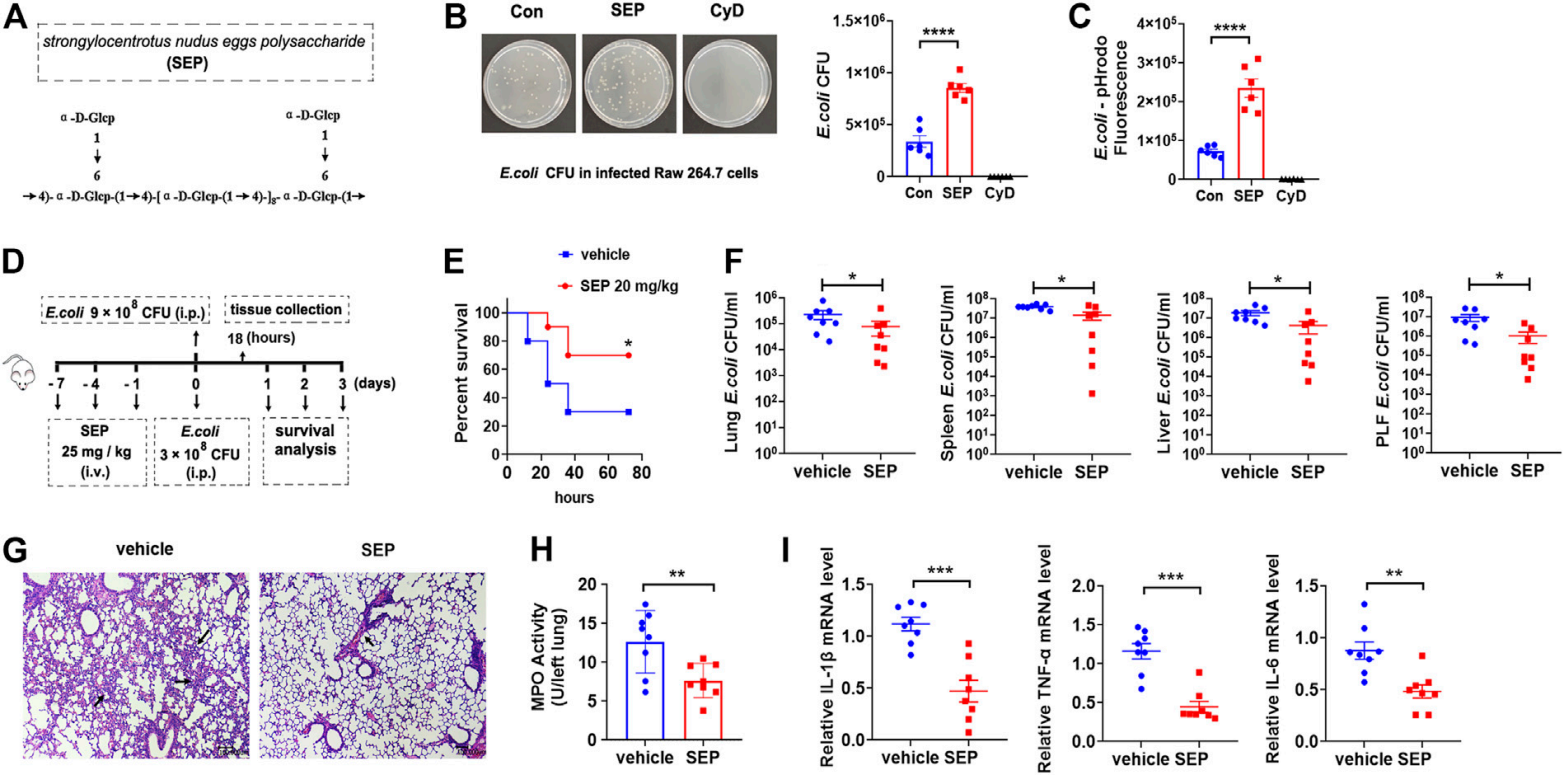
SEP protects against *E. coli* infection by enhancing host defense. **(A)** Molecular structure of SEP. **(B)** The effect of SEP on macrophages phagocytosis of live *E. coli* by gentamicin protection assay. RAW264.7 cells were administrated with Cyt D (10 μM) for 2 h prior to *E. coli* infection. **(C)** The effect of SEP on macrophages phagocytosis by fluorescence intensity. RAW264.7 cells were fed with pHrodo-conjugated inactivated *E. coli* bioparticles for 1 h and phagocytosis was quantified by the fluorescence. **(D)** The experimental design of *E. coli* infection and SEP treatment in mice. Mice were pre-treated with SEP (25 mg/kg) or vehicle (saline) by i. v. every 3 days for three times, and infected with *E. coli* (9 × 10^8^ CFU per mouse, i. p.) on the eighth day. Then the survival of mice **(E)** were evaluated. (*n* = 10). **(F)** Mice were pre-treated with SEP (25 mg/kg, i. v.) and infected with *E. coli* (3 × 10^8^ CFU per mouse, i. p.) on day eight, then sacrificed at 18 h post-infection. (*n* = 8). CFU of *E. coli* in tissue homogenates of lung, spleen, liver, and peritoneal lavage fluid were detected. **(G)** Lung sections were stained with H&E. **(H)** Myeloperoxidase (MPO) value were detected in the lung tissue. **(I)** The expression of IL-1β, TNF-α and IL-6 in the lungs were measured by ELISA. All data are represented as mean ± SEM acquired in triplicate determinations. *p < 0.05, **p < 0.01, ***p < 0.001.

Macrophages are important innate immune cells. The phagocytosis of bacteria by professional phagocytes, such as macrophages, is key to their microbicidal function. To explore whether the phagocytosis of live *E. coli* could be strengthened by SEP (Figure 1A), the gentamicin protection assay was employed (Sharma and Puhar, 2019) on RAW264.7 macrophages. Results showed that the number of intracellular live bacteria was effectively increased by SEP, but significantly decreased by Cytochalasin D (Cyt D), an F-actin polymerization inhibitor served as negative control (Figure 1B), suggesting the phagocytosis of live bacteria can be enhanced by SEP. The pHrodo (pH-reporter fluorescent dye) only shows strong fluorescence in low-pH environments, such as in phagosomes of mammalian cells, and has been validated as an effective strategy for discrimination between surface-bound and internalized particles (Sharma and Puhar, 2019). Thus, RAW264.7 macrophages were incubated with pHrodo-conjugated inactivated *E. coli,* and fluorescence following bacterial uptake was read on a plate reader. As shown in Figure 1C, the fluorescence was significantly increased in the SEP pretreated RAW264.7 cells, while dramatically decreased with the treatment of Cyt D. These data suggested that SEP promoted bacterial clearance by macrophage phagocytosis.

*E. coli* is a leading cause of lethal bacteremia. To investigate the contribution of SEP to host defense against bacterial, the model of *E. coli* induced sepsis was introduced, which was ordinarily accompanied by severe lung inflammation and bacterial burden in different organs. Briefly, mice were pre-treated with SEP (25 mg/kg, i. v.) or vehicle (saline) every 3 days for three times, then administered *E. coli* (9 × 10^8^ CFU, i. p.) to determine the effect of SEP on mouse mortality (Figure 1D). Remarkably, SEP decreased the mortality induced by bacterial infection in 3 days (Figure 1E), showing definite protection of SEP against *E. coli* infection. In view of SEP not killing *E. coli* directly, the hypothesis was proposed that SEP might contribute to bacterial clearance by innate host defense. To make sure all mice were alive during infection, a lower dose of *E. coli* (3 × 10^8^ CFU, i. p.) was adopted and the mice were sacrificed at 18 h post-infection. Compared with the vehicle group, SEP pre-treated mice displayed less *E. coli* dissemination to the lung, liver and spleen, and lower *E. coli* burdens in the peritoneal lavage fluid (Figure 1F), suggesting that the innate host defense may be enhanced by SEP. Assessment of inflammatory status (H&E staining) showed alveolar congestion accompanied by a large number of inflammatory cells infiltration in the vehicle group mice, while slight and less in the SEP group (Figure 1G). Moreover, the inflammatory state alleviated by SEP was also reflected by a lower level of lung myeloperoxidase (MPO), a marker of neutrophilic inflammation following bacterial infection (Figure 1H). Furthermore, inflammatory cytokines such as IL-1β, TNF-α and IL-6 in the peritoneal macrophage from SEP pre-treated mice were significantly decreased compared to the vehicle group (Figure 1I). Taken together, these observations demonstrated that the strengthened host defense against bacterial infection might be due to the enhanced macrophage phagocytosis by SEP.

### *Strongylocentrotus nudus* eggs polysaccharide Promotes Macrophages Phagocytosis *via* TLR4

**FIGURE 2:**
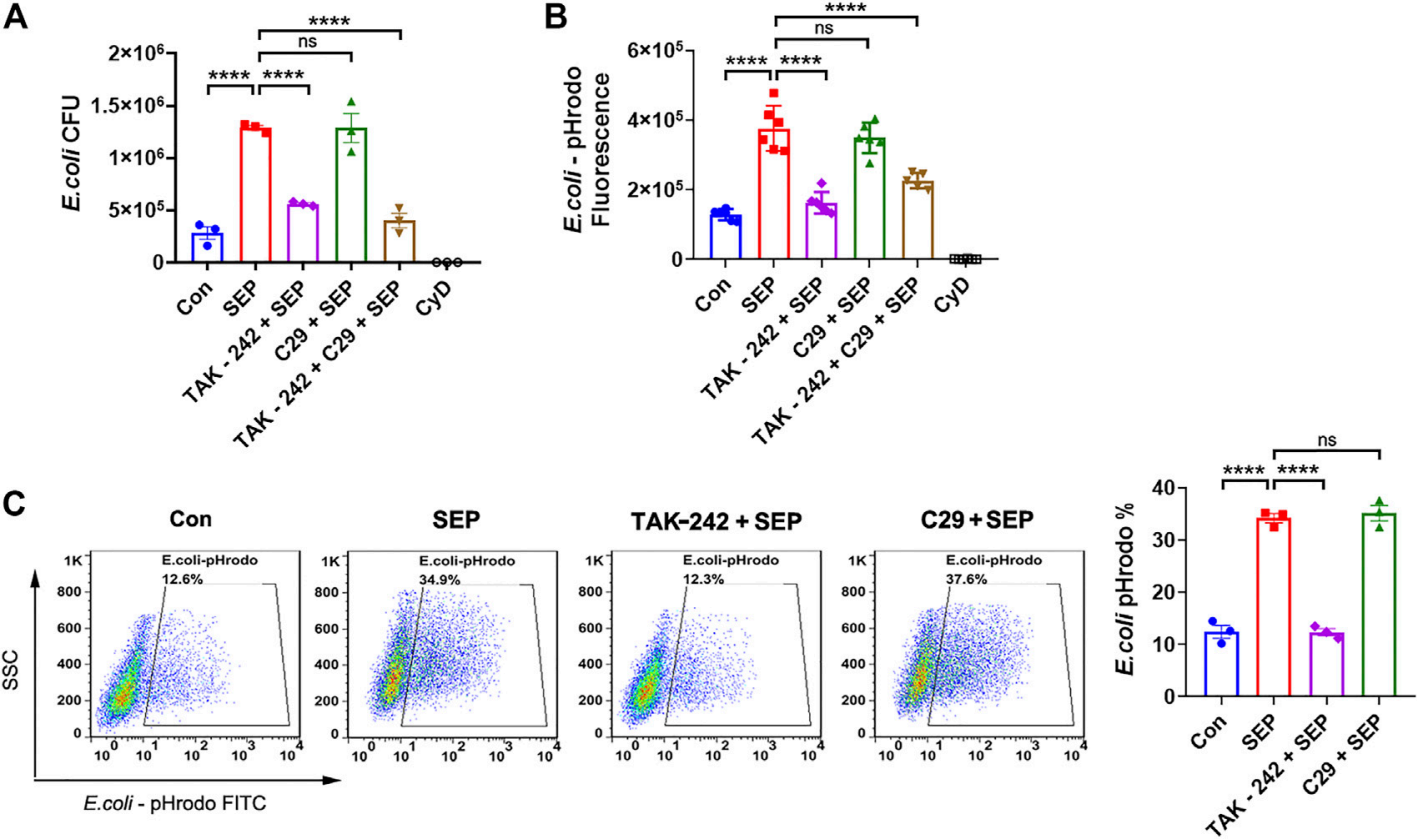
SEP promotes macrophages phagocytosis *via* TLR4. RAW264.7 cells were pre-treated with TLR2 inhibitor C29 (50 μM) or TLR4 inhibitor TAK-242 (20 μM) for 2 h, then stimulated with SEP (100 μg/ml) for another 24 h. **(A)** Phagocytosis of live *E. coli* by macrophages were measured by gentamicin protection assay. **(B)** Phagocytosis of pHrodo-*E. coli* by macrophages were quantified by fluorescence intensity. **(C)** The positive cell rates for phagocytosis of pHrodo-*E. coli* by macrophage were detected by flow cytometry. All data are represented as mean ± SEM acquired in triplicate determinations. ****p < 0.0001. ns, not significant.

Our previous work had reported that SEP activated NK cells to kill tumor cells *via* both TLR2 and TLR4 signaling pathways (Wang et al., 2011; Ke et al., 2014). Meanwhile, it is well established that TLR2 and TLR4 signaling promote macrophage phagocytosis (Fang et al., 2014; Lv et al., 2017). To test whether TLR2 and/or TLR4 signaling are involved in the SEP induced phagocytosis, RAW264.7 cells were pre-treated with TLR2 inhibitor C29 or TLR4 inhibitor TAK-242 for 2 h and then stimulated with SEP for another 24 h. Interestingly, TAK-242, but not C29, significantly reduced the numbers of SEP induced intracellular live *E. coli* (Figure 2A) and abolished the intracellular fluorescence of pHrodo-conjugated *E. coli* induced by SEP (Figure 2B). Furthermore, the positive cell rates for phagocytosis of pHrodo-*E. coli* by macrophage were also analyzed by flow cytometry, and the results showed the percentage of SEP induced phagocytosis of pHrodo-*E. coli* by macrophage were significantly decreased by TAK-242 (Figure 2C). In summary, these data indicated that TLR4, rather than TLR2, mediated SEP induced macrophages phagocytosis.

### TLR4/STAT3 Signaling is Involved in SEP Induced Phagocytosis

**FIGURE 3:**
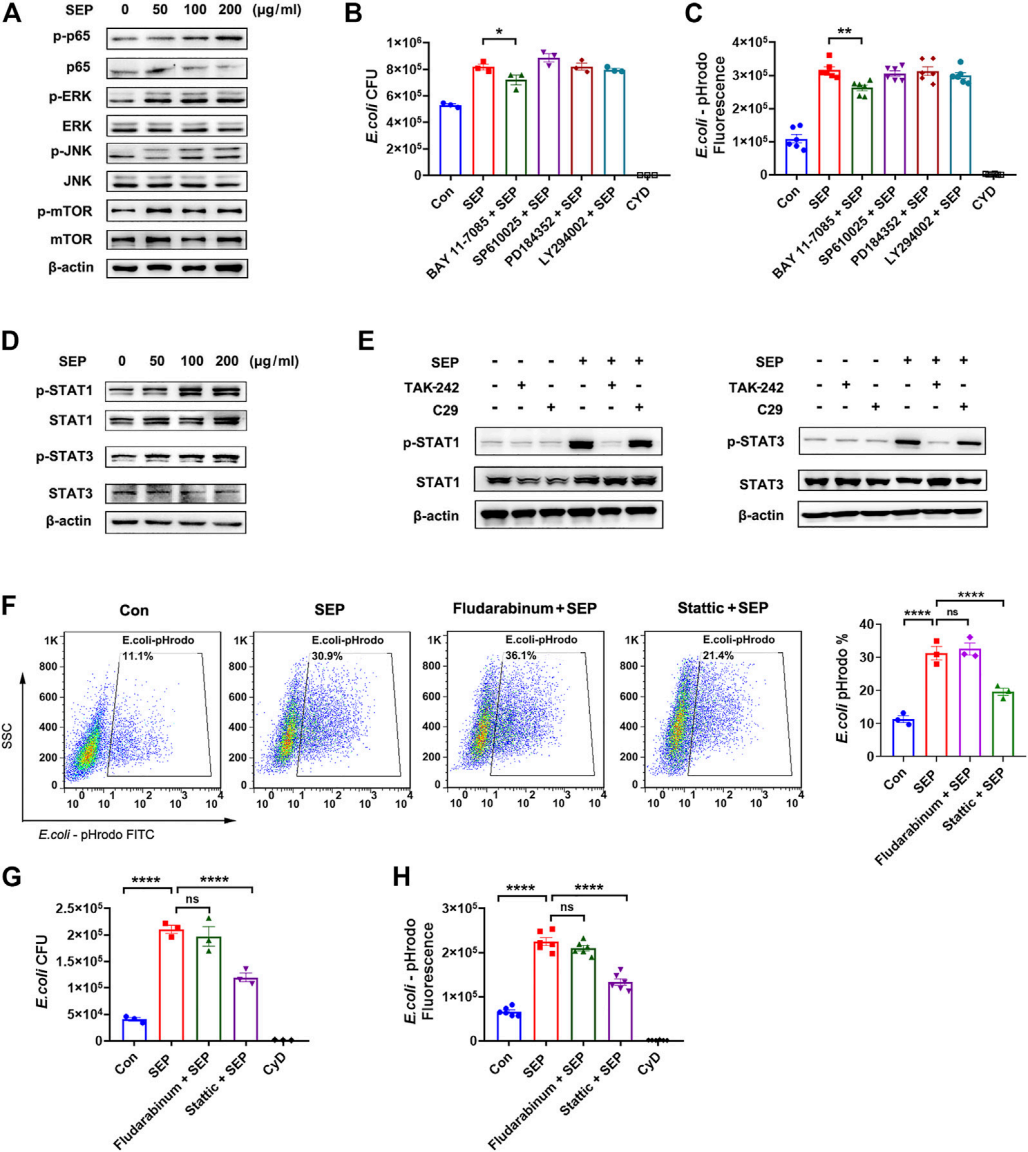
TLR4/STAT3 signaling is involved in SEP induced phagocytosis. **(A)** The effects of SEP on protein levels of p-p65, p-ERK, p-JNK and p-mTOR were determined by western blot. **(B)** The effects of TLR4 signaling pathway inhibitors on phagocytosis of live *E. coli* were measured by gentamicin protection assay. RAW264.7 cells were pre-treated with NF-κB inhibitor BAY 11-7085 (2 μM), or JNK inhibitor SP610025 (5 μM), or MEK1/2 inhibitor PD184352 (5 μM), or PI3K inhibitor LY294002 (5 μM) for 2 h prior to SEP treatment. **(C)** Phagocytosis of pHrodo-*E. coli* by macrophages were quantified by fluorescence intensity. **(D, E)** The effects of SEP on protein levels of p-STAT1, p-STAT3 were determined by western blot. The effects of Stattic (5 μM) or Fludarabine (20 μM) on phagocytosis of pHrodo-*E. coli* were measured by flow cytometry **(F)**, or phagocytosis of live *E. coli* by gentamicin protection assay **(G)**, or phagocytosis of pHrodo-*E. coli* by fluorescence intensity **(H)**. All data are represented as mean ± SEM acquired in triplicate determinations. *p < 0.05, **p < 0.01, ****p < 0.0001.

It is well established that the downstreams of TLR4 signal including NF-κB, MAPK, PI3K, STAT1/3 are involved in phagocytosis (Fang et al., 2017; Huynh et al., 2020; Yang et al., 2021). As shown in Figure 3A, SEP significantly up-regulated the phosphorylation levels of NF-κB p65, ERK, JNK and mTOR in RAW264.7 cells, suggesting that TLR4 signaling pathway indeed was activated by SEP in macrophage. To explore which signal mediates the role of SEP, several specific inhibitors were used to assess macrophages phagocytosis. The results showed that compared to the SEP, only BAY 11-7085 (NF-κB inhibitor) slightly reduced the number of intracellular live bacteria and decreased SEP induced intracellular fluorescence of pHrodo-*E. coli* (Figures 3B,C), suggesting that the role of NF-κB signal is limited. Reports showed that STAT3 is important for defense against bacterial infection (Kuuliala et al., 2018; Lei et al., 2021). The results showed that SEP significantly up-regulated the phosphorylation levels of STAT1 and STAT3 (Figure 3D). Moreover, TLR4 inhibitor TAK-242, but not TLR2 inhibitor C29, inhibited SEP induced activation of STAT1 and STAT3 (Figure 3E). Of note, STAT3 inhibitor Stattic, but not STAT1 inhibitor Fludarabine, significantly abolished SEP induced positive cell rates for phagocytosis of pHrodo-*E. coli* by macrophage, the live intracellular bacterial numbers and the intracellular fluorescence (Figures 3F–H). Taken together, these results suggested that TLR4/STAT3 signaling was critical for SEP induced macrophages phagocytosis.

### CD64 is Responsible for SEP Induced Phagocytosis *via* TLR4/STAT3 Signals

**FIGURE 4:**
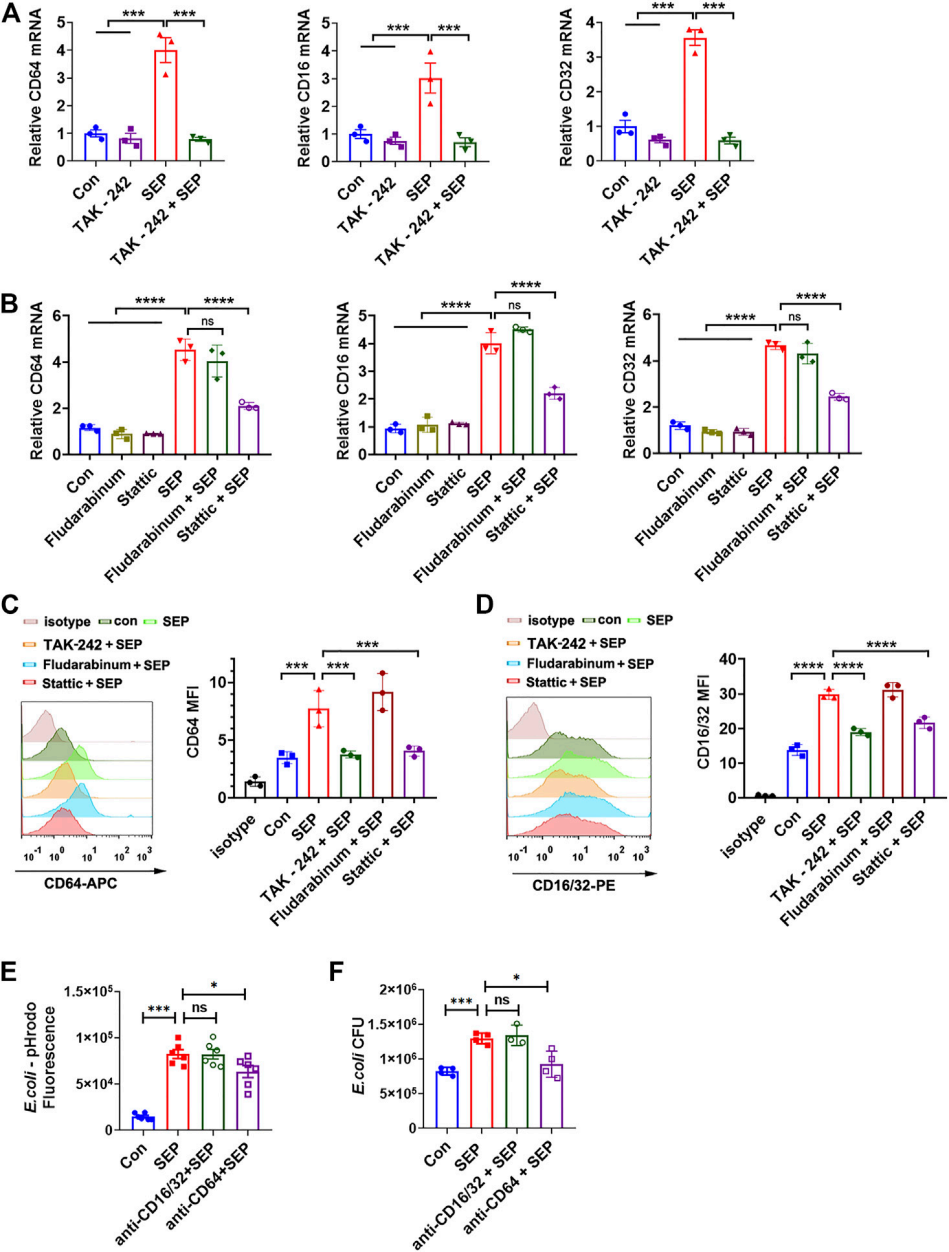
CD64 is responsible for SEP induced phagocytosis *via* TLR4/STAT3 signals. **(A,B)** The effects of SEP on the relative mRNA expression of CD64, CD32 and CD16 were determined by q-PCR. **(C,D)** The effects of SEP on the protein levels of CD64 and CD16/32 were determined by flow cytometry. **(E)** RAW264.7 cells were pre-treated with SEP (100 μg/ml) for 24 h, and followed by incubation with 2 μg/ml anti-CD64 or anti-CD16/32 blocking antibodies for 1 h prior to pHrodo-*E. coli* infection. Then macrophage phagocytosis was quantified by the fluorescence. **(F)** The effects of blocking CD64 or anti-CD16/32 on phagocytosis of live *E. coli* by macrophages were measured by gentamicin protection assay. All data are represented as mean ± SEM acquired in triplicate determinations. *p < 0.05, ***p < 0.001,****p < 0.0001. ns, not significant.

The recognition and phagocytosis of bacterial pathogens by phagocytes depend on phagocytic receptors on the cell membrane. FcγRs are essential phagocytic receptors for macrophages to respond to bacteria (Aderem and Underhill, 1999; Mittal et al., 2010). Therefore, the expression of CD64 (FcγRI), CD32 (FcγRII) and CD16 (FcγRIII) were assessed in RAW264.7 cells with various stimuli. As shown in Figure 4A, SEP significantly increased the mRNA expression levels of CD64, CD32 and CD16, while inhibition of TLR4 or STAT3 effectively decreased the transcription of FcγRs induced by SEP, but not STAT1 (Figures 4A,B). Moreover, the protein expression level of FcγRs were consistent with the mRNA levels under the corresponding stimulation (Figures 4C,D). To determine the role of FcγRs in SEP induced *E. coli* uptake, blocking antibodies against mice CD64 and CD16/32 were used on macrophage. The results showed that blockage of CD16/32 had no effect on SEP induced intracellular fluorescence of pHrodo-*E. coli* and the number of intracellular live bacteria (Figures 4E,F). However, blockade of CD64 did cause a significant decrease in phagocytosis of *E. coli* (Figures 4E,F). These results indicated that SEP up-regulated FcγRs expression by the activated TLR4/STAT3 axis signaling, and CD64 mediated the uptake and phagocytosis of *E. coli.*

### SEP Promotes Formation of Filopodia *via* TLR4/STAT3 Axis

**FIGURE 5:**
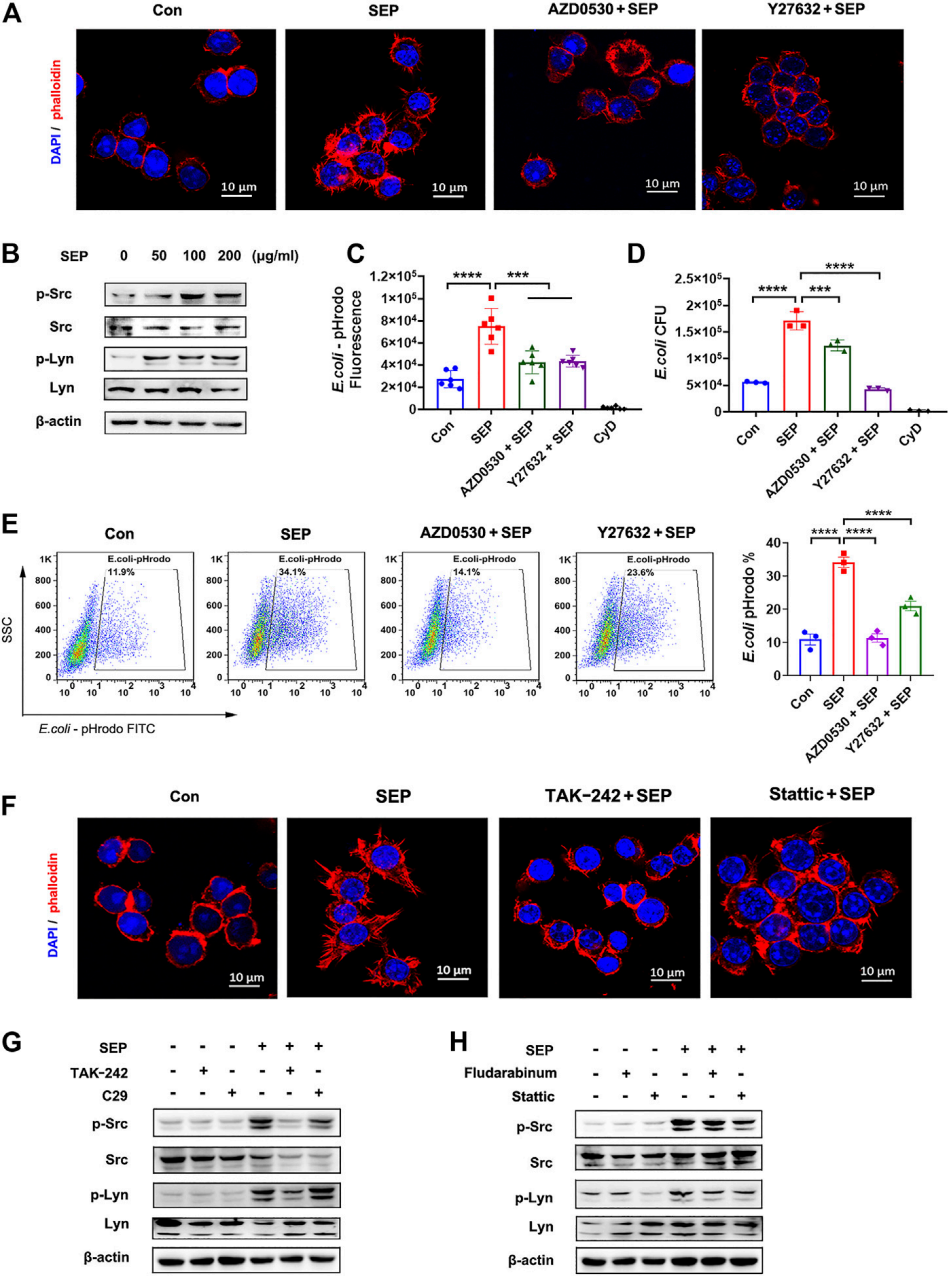
SEP promotes filopodia formation *via* TLR4/STAT3 axis. **(A)** The effects of SEP on the filopodia formation of macrophages. RAW264.7 cells were administrated with Src family inhibitor AZD0530 (10 μM) or ROCK1 inhibitor Y-27632 (20 μM) for 2 h prior to *E. coli* infection. The cellular filamentous actin were stained with rhodamine phalloidin and the cellular DNA with DAPI. **(B)** The effects of SEP on protein levels of p-Src and p-Lyn were determined by western blot. **(C)** The effects of AZD0530 or Y-27632 on SEP induced phagocytosis of pHrodo-*E. coli* were measured by fluorescence intensity, **(D)** or phagocytosis of live *E. coli* by gentamicin protection assay, or **(E)** phagocytosis of pHrodo-*E. coli* by flow cytometry. **(F)** The effects of TAK-242 or Stattic on SEP induced filopodia formation. **(G,H)** The effects of TAK-242, or C29, or Stattic, or Fludarabine on SEP induced protein levels of p-Src and p-Lyn were determined by western blot. ***p < 0.001, ****p < 0.0001.

Filopodia formation, the process of which includes F-actin polymerization and cytoskeleton remodeling, is thought to be critical for macrophages to perform bacterial phagocytosis (Li et al., 2017). After recognition of pathogens, FcγRs activates Src family of tyrosine kinases, such as Src and Lyn, and subsequently followed by the activation of Ras, Rac and ROCK, inducing actin cytoskeleton re-arrangement to form Filopodia (Kress et al., 2007; Rougerie et al., 2013). To visualize the effect of SEP on filopodia formation, RAW264.7 cells were stimulated with SEP for 24 h, then F-actin was stained with phalloidin and observed using a confocal microscope. As shown in Figure 5A, SEP promoted the formation of F-actin spikes, suggesting that SEP induced the formation of filopodia. To determine whether Src and Lyn are involved in the role of SEP on filopodia formation and macrophage phagocytosis, RAW264.7 cells were administrated with pharmacological inhibitors targeting Src family (AZD0530) and ROCK1 (Y-27632) for 2 h prior to *E. coli* infection. As shown in Figure 5B, the phosphorylation levels of Src and Lyn were significantly up-regulated in response to SEP treatment, while Src and ROCK1 inhibitors decreased the macrophages phagocytosis to *E. coli* induced by SEP (Figures 5C–E). In addition, inhibition of Src or ROCK1 also significantly reduced SEP induced filopodia formation (Figure 5A), suggesting that SEP induced phagocytosis to *E. coli* depended on the activation of Src.

To determine whether TLR4/STAT3 signaling is involved in SEP induced filopodia formation, TAK-242 or Stattic was used. As shown in Figure 5F, SEP induced filopodia formation was almost reversed by TAK-242 or Stattic, suggesting that SEP induced filopodia formation is dependent on TLR4/STAT3 axis. To further investigate whether TLR4 or STAT3 is involved in SEP induced Src and Lyn activation, TLR4 or STAT3 inhibitors were used before SEP treatment. As shown in Figures 5G,H, inhibition of TLR4 or STAT3 resulted in a significant decrease in the phosphorylation levels of Src and Lyn in RAW264.7 cells with SEP treatment. Collectively, these results indicated that SEP promoted the activities of Src and Lyn through TLR4/STAT3 signals, which led to the enhanced filopodia formation and increased phagocytosis of bacteria.

### Inhibition of TLR4 Retardes the Protection of SEP Against *E.coli* Infection

**FIGURE 6:**
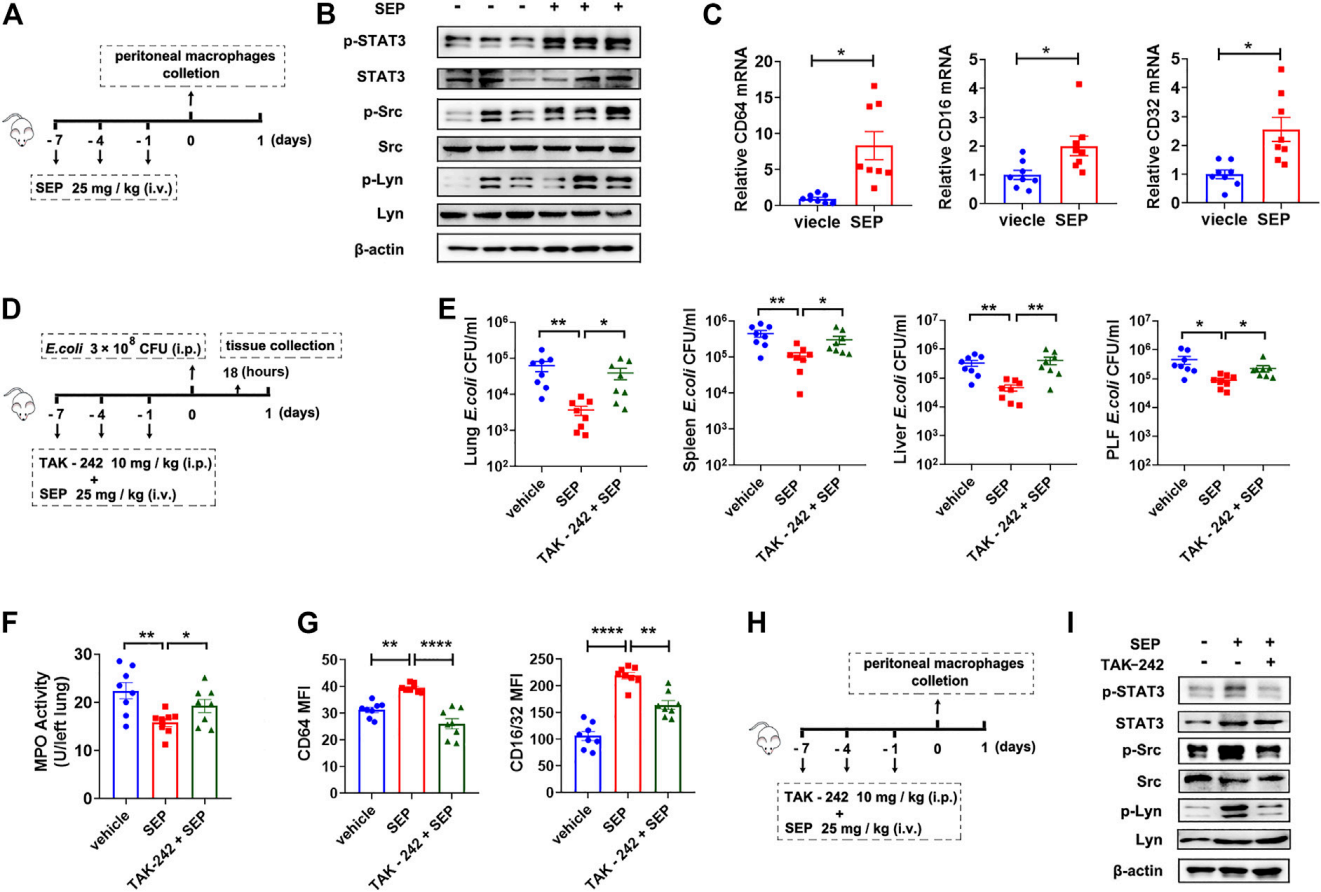
Inhibition of TLR4 abolishes the protection of SEP against *E. coli* infection. **(A)** The experimental design of SEP administration in mice. Mice were treated with SEP (25 mg/kg) or vehicle (saline) by i. v. every 3 days for three times, and sacrificed on the eighth day. The total protein and mRNA were extracted from mice peritoneal macrophages. **(B)** The protein levels of p-STAT3, p-Src and p-Lyn in the peritoneal macrophages were determined by western blot. **(C)** The relative mRNA levels of CD64, CD32 and CD16 in the peritoneal macrophages were determined by q-PCR. **(D)** The experimental design of TAK-242 combined with SEP administration in mice infection model. The TAK-242 group mice were pre-treated with TAK-242 (10 mg/kg, i. p.) 2 h before administration of SEP, once every 3 days for three times. Then mice were further challenged with *E. coli* (3 × 10^8^ CFU, i. p) on day eight and sacrificed at 18 h post-infection. (*n* = 8). **(E)** CFU of *E. coli* was detected in tissue homogenates of lung, spleen, liver, and peritoneal lavage fluid. **(F)** MPO value in the lung tissue. **(G)** The protein levels of CD64 and CD16/32 in the peritoneal macrophages were determined by flow cytometry. **(H)** The experimental design of TAK-242 combined with SEP administration in mice. The TAK-242 group mice were pre-treated with TAK-242 (10 mg/kg, i. p.) 2 h before administration of SEP, once every 3 days for three times, and sacrificed on the eighth day. **(I)** The protein levels of p-STAT3, p-Src and p-Lyn in the peritoneal macrophages were determined by western blot. All data are represented as mean ± SEM acquired in triplicate determinations. *p < 0.05, **p < 0.01, ****p < 0.0001.

To ascertain the effects of SEP on TLR4 signaling *in vivo*, mice were treated with SEP and sacrificed (Figure 6A **)**, then the activities of the key components were detected. In line with the effect of SEP *in vitro*, the phosphorylation levels of STAT3, Src and Lyn were significantly up-regulated in the peritoneal macrophages of SEP pre-treated mice (Figure 6B), as well as the mRNA expression level of FcγRs (Figure 6C). To further confirm that TLR4 signaling is involved in SEP afforded bacterial clearance *in vivo*, TLR4 inhibitor TAK-242 was introduced. Mice were pre-treated with TAK-242 (10 mg/kg, i. p.) and administered with SEP (25 mg/kg, i. v.) 2 h later, once every 3 days for three times. Then mice were further challenged with *E. coli* (3 × 10^8^ CFU, i. p) and sacrificed at 18 h post-infection (Figure 6D). As expected, TAK-242 increased the counts of bacteria in the lung, spleen, liver and peritoneal lavage fluid compared to SEP pre-treated mice (Figure 6E), indicating that the inhibition of TLR4 retarded the bacterial clearance accelerated by SEP. Moreover, the decreased lung myeloperoxidase (MPO) level in SEP pre-treated mice was also restored by TAK-242 (Figure 6F). Consistent with the findings *in vitro*, the up-regulated protein levels of FcγRs in peritoneal macrophages from SEP pre-treated mice were also significantly reversed by TAK-242 (Figure 6G), as well as the phosphorylation levels of STAT3, Src and Lyn (Figures 6H,I). These results indicated that the activated TLR4 signaling in macrophages playsed a key role in the anti-infection of SEP.

## Discussion

Boosting innate host defense has gained substantial attention on antimicrobial therapy due to the prevalence of antibiotic-resistant bacteria over recent years (Akira et al., 2006; Hamill et al., 2008; Kaufmann et al., 2017). Since the mechanisms of innate host defense are independent of the bactericidal pathways of antibiotics, host directed therapy by enhancing innate immunity may provide therapeutic strategies against bacterial infection regardless of their antibiotic resistance status (Si-Tahar et al., 2009; Kaufmann et al., 2017). Therefore, this study focused on exploiting the effect of SEP on innate host defense against bacterial infections.

TLR4, a major pattern recognition receptor of innate immunity, regulates innate host defense *via* detecting invasive pathogens and triggering inflammatory responses (Hernandez et al., 2019). TLR4 responds to LPS facilitating elimination of invading bacteria during infection (Poltorak et al., 1998b), but also immediately triggers exaggerated pro-inflammatory responses even leading to sepsis or death (Poltorak et al., 1998a). On one hand, TLR4 inhibition was once considered to be an innovative therapeutic approach targeting pathogen infection, such as sepsis (Bagheri et al., 2014; Romerio and Peri, 2020). However, two TLR4 inhibitors, Eritoran and Tak-242 failed to improve the survival of septic patients in the phase III clinical trials as antisepsis agents (Rice et al., 2010; Anwar et al., 2019). Moreover, mice without functional TLR4 showed a higher mortality and impaired bacterial clearance in polymicrobial sepsis or bacteria infection (Fujiwara et al., 2013; Zhang et al., 2014; Gugliandolo et al., 2019). On the other hand, LPS as an immunomodulator to enhance macrophage phagocytosis and bacterial clearance had been largely abandoned in the clinical application due to its inflammation related toxicity and macrophage pyroptosis (Rietschel et al., 1994; Hernandez et al., 2019). Interestingly, TLR4-overexpression transgenic mice and sheep exhibited a survival advantage and improved bacterial phagocytosis during bacterial infection (Bihl et al., 2003; Wan et al., 2018). Notably, monophosphoryl lipid A (MPLA), a mild TLR4 agonist, provides a survival benefit to an array of pathogenic bacteria by enhancing macrophage bacterial clearance and attenuating inflammation (Romero et al., 2011; Fensterheim et al., 2018; Fukuda et al., 2020; Owen et al., 2021). Thus, the appropriate threshold level for TLR4 activation is beneficial to anti-bacterial infection.

Polysaccharide SEP is extracted from seafood *Strongylocentrotus nudus* eggs with high nutritional value. It has been reported that SEP exhibited immunomodulatory effects on T cells, NK cells, B cells and macrophages without any adverse reactions (Liu et al., 2008; Wang et al., 2011; Ke et al., 2014; Xie et al., 2017; Hu et al., 2018; Xie et al., 2019). In this study, SEP exhibited strong protection against *E. coli* induced mortality (Figure 1E) and lower *E. coli* burdens in the lung, liver, spleen and the peritoneal lavage fluid (Figure 1F). Moreover, SEP attenuated the MPO level in the lung and inflammatory cytokines IL-1β, TNF-α, IL-6 in the peritoneal macrophage during infection (Figures 1H,I). TLR4 signaling was activated in the peritoneal macrophages of mice pre-treated with SEP, while TLR4 inhibition by TAK-242 retarded bacterial clearance and increased the lung MPO level (Figures 6E,F). All these data above suggested that SEP should be a mild TLR4 agonist protecting against *E. coli* infection rather than organ injury. Of note, the mice involved in this study only experienced controlled infection rather than secondary infection. In fact, secondary infection is more common in critically ill patients, such as burn patients suffered from antibiotic resistant infections. The impact of SEP on postinjury infection needs further exploration.

FcγRs are phagocytic receptors necessary for macrophages to recognize and phagocytize bacteria. Here, SEP up-regulated the expression levels of FcγRs, and CD64 was the effective FcγR for SEP enhanced phagocytosis, not CD16 or CD32 (Figures 4E,F). It was reported that the level of CD64 expression in macrophages of sepsis was higher in survivors (Danikas et al., 2008) and the high expression of CD64 in proinflammatory macrophages (M1) was responsible for the phagocytosis and killing of pathogen (Akinrinmade et al., 2017). Of note, CD64 is involved in positive regulation of protein tyrosine kinase to trigger cellular activation. Here, SEP promoted F-actin polymerization and filopodia formation (Figure 5A). Interestingly, the kinase activity of Src and Lyn, two critical factors to induce F-actin polymerization and filopodia formation, were enhanced by SEP through TLR4/STAT3 axis signaling (Figure 5). Although only *E. coli* was used in this study, the up-regulated levels of FcγRs and the enhanced filopodia formation in macrophage suggested that SEP might generally protect against various bacterial infections through the enhanced innate immunity. It is meaningful to explore whether SEP has protective effect against Gram-positive bacterial or polymicrobial infection.

The TLR4 signaling plays an important role in endotoxin tolerance (Vergadi et al., 2018). Endotoxin tolerance attenuates inflammation, and confers resistance to a broad array of microbes infection including Gram positive or negative bacteria, and fungal pathogens as well as polymicrobial sepsis (Vergadi et al., 2018). Although endotoxin tolerance protects the host by limiting excessive inflammatory responses, it compromises the ability of macrophages and neutrophils to counteract secondary infections (Lopez-Collazo and del Fresno, 2013; Vergadi et al., 2018), leading to immunosuppression and mortality in patients with sepsis or noninfectious systemic inflammatory response syndrome (SIRS) (Lopez-Collazo and del Fresno, 2013). It was reported that MPLA can induce endotoxin tolerance in endothelial cells, attenuate LPS induced cytokine production and protect against LPS induced endothelial dysfunction (Romero et al., 2011; Stark et al., 2015). In addition, depletion of macrophages or neutrophils resulted in the loss of MPLA induced survival benefit (Fensterheim et al., 2018; Owen et al., 2021). In this study, SEP activated TLR4 signaling pathway and markedly protected against *E. coli* infection, improved bacterial clearance and attenuated systemic cytokine production. Further study is needed to detect whether SEP could induce endotoxin tolerance.

In summary, SEP enhanced innate host defense through regulation of macrophages in phagocytic activity and bacterial clearance, improved survival and outcomes in *E. coli* infection. The underlying mechanism of SEP was involved in the increased activity of TLR4/STAT3/CD64 signaling in macrophages, and provided some evidence for its potential clinical development as immunoprophylaxis or adjunct antimicrobial agents for infection.

## Data Availability

The original contributions presented in the study are included in the article/Supplementary Material, further inquiries can be directed to the corresponding authors.
